# Editorial: Implementation of digital health intervention on mobile devices to support cardiovascular disease healthcare among public health

**DOI:** 10.3389/fcvm.2024.1379138

**Published:** 2024-02-13

**Authors:** Han Feng, Lingzhi Li, Chaoran Hu, Hua He, Nassir Marrouche, Xiang Li

**Affiliations:** ^1^ Tulane Research Innovation for Arrhythmia Discovery, Tulane University School of Medicine, New Orleans, LA, United States; ^2^ Division of Nephrology, Kidney Research Institute, West China Hospital of Sichuan University, Chengdu, China; ^3^ Eli Lilly and Company, Indianapolis, IN, United States; ^4^ Department of Epidemiology, School of Public Health and Tropical Medicine, Tulane University, New Orleans, LA, United States; ^5^ Department of Biostatistics and Data Science, School of Public Health and Tropical Medicine, Tulane University, New Orleans, LA, United States; ^6^ Division of Endocrinology, Diabetes, and Metabolism, Department of Medicine, College of Medicine, University of Illinois Chicago, Chicago, IL, United States

**Keywords:** digital health, AI, mobile devices, cardiovascular disease, public health

**Editorial on the Research Topic** Implementation of digital health intervention on mobile devices to support cardiovascular disease healthcare among public health

This Research Topic collection, entitled “Implementation of Digital Health Intervention on Mobile Devices to Support Cardiovascular Disease Healthcare” in Frontiers in Cardiovascular Medicine, aims to aggregate seminal manuscripts that exemplify the confluence of digital health within the realm of cardiovascular disease healthcare. The editorial board has meticulously curated this collection to highlight scholarly works of high quality, with an emphasis on propelling the understanding of digital health integration into routine cardiovascular care or providing substantive evidence for the potential expansion of digital health practices. This anthology not only showcases the caliber and scope of research featured in this segment but also reflects the global diversity of our scholarly community. The editor's narrative that follows offers a comprehensive overview of the articles included, highlighting their academic significance and potential impact on public health.

Digital health has emerged as a transformative force, accelerating the velocity of healthcare service delivery. Leveraging the pervasive nature of mobile applications, it facilitates a cost-effective and easily accessible healthcare service, with the potential to substantially reduce the number of underserved populations. Moreover, mobile devices have great potential to carry out effective instruments for health screening and monitoring that have been shown in some other studies (1, 2). In this collection, we present two trial papers and one protocol paper that provide illuminating examples of how mobile technology can be harnessed to enhance the quality and reach of cardiovascular healthcare services. We also include two methodological papers that pave the way for the future of mobile health diagnostics. These papers delve into the development of advanced machine learning and AI algorithms designed to enhance screening and diagnostic procedures, which mobile devices may come to incorporate in forthcoming iterations (Table 1).

**Table 1:** Metrics of the articles published in implementation of digital health intervention on mobile devices to support cardiovascular disease healthcare among public health.

Title of manuscript	First author	Country	Views^a^
“Own doctor” presence in a web-based lifestyle intervention for adults with obesity and hypertension: a randomized controlled trial	Pedro Múzquiz-Barberá	Spain	2,173
Effect of a monitored home-based exercise program combined with a behavior change intervention and a smartphone app on walking distances and quality of life in adults with peripheral arterial disease: the WalkingPad randomized clinical trial	Ivone Silva	Portugal	659
The effect of hospital-to-home transitional care using a digital messaging application on the health outcomes of patients undergoing CABG and their family caregivers: a randomized controlled trial study protocol	Maryam Maleki	Iran	575
Improvement of a prediction model for heart failure survival through explainable artificial intelligence	Pedro A. Moreno-Sánchez	Finland	1,669
Machine learning-based detection of cardiovascular disease using ECG signals: performance vs. complexity	Huy Pham	Russia	1,900

^a^ Until January 4, 2024.

“Own doctor” presence in a web-based lifestyle intervention for adults with obesity and hypertension: A randomized controlled trial (Múzquiz-Barberá et al.).

This trial aimed to investigate whether patients' outcomes in a web-based intervention program are influenced by the presence of their own doctor vs. an unknown doctor. This study is novel as it is the first to analyze this factor within online interventions aimed at promoting healthy lifestyles among adults with obesity and hypertension. The research found no significant additional benefits from the inclusion of patients' own doctors in the audiovisual content of the intervention, suggesting that the identity of the doctor presenting the intervention might not be a critical factor in the effectiveness of web-based health counseling.

Effect of a monitored home-based exercise program combined with a behavior change intervention and a smartphone app on walking distances and quality of life in adults with peripheral arterial disease: the WalkingPad randomized clinical trial (Silva et al.).

This work introduces an innovative home-based exercise program supported by a smartphone application. The novelty lies in combining exercise with behavioral interventions and digital tracking to enhance walking distances and quality of life in peripheral arterial disease patients. It demonstrates the effectiveness of the WalkingPad app in improving maximal walking distances over 6 months, particularly in patients without severe anxiety symptoms, marking a significant contribution to digital health interventions.

The effect of hospital-to-home transitional care using a digital messaging application on the health outcomes of patients undergoing CABG and their family caregivers: a randomized controlled trial study protocol (Maleki et al.).

This study is groundbreaking as it will be the first RCT to evaluate the effectiveness of a digital messaging app in transitional care for CABG patients and caregivers. It emphasizes a novel person-centered care approach, anticipating improved self-efficacy, quality of life, and reduced caregiver burden, potentially revolutionizing post-discharge care protocols.

Improvement of a prediction model for heart failure survival through explainable artificial intelligence (Moreno-Sánchez).

This manuscript stands out for its novel approach to enhancing the interpretability of machine learning models in a clinical setting. The innovation of this work lies in its explainability approach, which is applied to both the classification and survival analysis prediction models for HF survival. This methodology enables healthcare professionals to better understand and interpret the models' outcomes, facilitating early identification of critical health changes in patients using a concise set of indicators. The use of this approach could significantly improve clinical decision-making by allowing clinicians to focus on treating the most relevant features to potentially prevent adverse outcomes.

Machine learning-based detection of cardiovascular disease using ECG signals: performance vs. complexity (Pham et al.).

This article brings forth several novel approaches to the utilization of machine learning algorithms for the detection of cardiovascular diseases through ECG signal analysis. The novelty in this work lies in its critical examination of the balance between the performance of machine learning models and their computational complexity, presenting a new perspective on optimizing the efficiency and effectiveness of ECG-based diagnostic tools. This contribution is significant as it addresses the practical constraints of deploying advanced AI in real-world clinical environments, where resources and processing power may be limited.

Collectively, all the work mentioned above illuminates the practicalities of digital health interventions in real-world clinical scenarios and supports the scaffolding of innovative digital health implementations. We hope this research topic collection provides a good example of how embracing digital health improves cardiovascular health care and inspires future research.
